# Global requirements for manufacturing and validation of clinical grade extracellular vesicles

**DOI:** 10.1016/j.jlb.2024.100278

**Published:** 2024-11-20

**Authors:** Abhimanyu Thakur, Deepika Rai

**Affiliations:** a Department of Neurosurgery, Massachusetts General Hospital, Harvard Medical School, Boston, MA, United States; b Smidt Heart Institute, Cedars-Sinai Medical Centre, Los Angeles, CA, United States

**Keywords:** Extracellular vesicles, Exosomes, Micro vesicles, Apoptotic bodies, Clinical grade product

## Abstract

Extracellular vesicles (EVs) are nanovesicles released from different cell types from biofluids such as blood, urine, and cerebrospinal fluid. They vary in size and biomarkers, and their biogenesis pathways allow them to be divided into three major types: exosomes, micro-vesicles, and apoptotic bodies. EVs have been studied in the context of diagnosis and therapeutic intervention of various pathological conditions such as cancer, neurodegenerative diseases, and pulmonary diseases. However, the production of EV-based therapeutics can be affected by the source, heterogeneity, or disease, raising questions about the manufacturing and validation of EVs of clinical grade and their scope regarding good manufacturing practice (GMP) in the industry. To address this, we have discussed the state-of-the-art requirements for EV production that must occur in a GMP-compliant environment with a reliable and traceable source. Additionally, EVs' homogeneity and the therapeutics' purity and stability must be analyzed and validated. Quality control measures must also be established to ensure the safety and efficacy of EVs. In conclusion, these considerations must be weighed carefully when manufacturing and validating EVs of clinical grade to ensure their safety and efficacy for therapeutic use.

## 1 Introduction

Extracellular Vesicles (EVs) are transportable lipid bilayer membrane-vesicles, mostly released from all cell types, and can be found in many biofluids, including blood and cerebrospinal fluid (CSF) [1,2]. The potential of EVs as a tool for identifying diagnostic biomarkers and using them as a carrier for therapeutic drugs, due to this reason, has been a point of interest from both academia and industry [[3], [4], [5], [6]]. This can be attributed to the reduced immunogenicity of EVs when compared to other delivery strategies, such as liposomes and viral vectors. Moreover, all these delivery strategies can cross biological barriers, such as the blood-brain barrier (BBB) [[7], [8], [9]].

The production and validation of clinical grade EVs is a critical component of drug development, as these vesicles are used for therapeutic and diagnostic purposes. Nearly half of the EV-related clinical trials focus on diagnostics, with cancer being the most common target. Additionally, EVs are explored for therapeutic use in respiratory illnesses, including COVID-19, highlighting their versatility and therapeutic promise [10,11]. To ensure the safety and efficacy of EVs for clinical solicitations, it is important to establish and adhere to global requirements for their manufacturing and validation. The first step in developing clinical-grade EVs is to ensure their quality and purity [12]. This can be accomplished through stringent manufacturing and process controls, including using standardized reagents and materials, implementing quality assurance oversight, and adhering to good manufacturing practices (GMPs) [13]. Additionally, EVs should be appropriately characterized to meet the desired specifications. These can include physicochemical and biological analyses, such as size distribution and protein content [14]. Next, it is important to validate the efficacy and safety of EVs for clinical applications. This can be done through pre-clinical and clinical testing, including immunogenicity, biodistribution, and pharmacokinetics measures. It is also important to ensure that EVs do not contain any toxic or contaminating agents that could harm patients [15]. Overall, the manufacturing and validation of clinical-grade EVs are essential steps in the drug development process. To ensure the safety and efficacy of EVs for clinical use, it is imperative to adhere to global requirements that include stringent manufacturing and process controls, appropriate characterization of EVs, and extensive pre-clinical and clinical testing [16,17].

Regulatory bodies such as the International Society for Extracellular Vesicles (ISEV), European Network on Micro-vesicles and Exosomes in Health and Disease (ME-HaD) have established regulations to promote the clinical implementation of exosomal therapy. These regulations provide detailed information about the standard operating procedures that must be followed during the collection, further processing, testing of the sample collected, quality assurance of the production, and using exosomes further for medical applications. This will ensure that EVs are used at the correct level for therapeutic purposes and that the full potential of EVs can be realized [18,19]. In the United States, no exosome products have been accepted by the Food and Drug Administration (FDA) for human use [20]. The FDA categorizes exosomes as a 351 product and requires research proving their safety, efficacy, purity, and effectiveness in treating the disorder [21]. Exosome-based therapies are being developed and must receive approval from regulatory bodies before entering clinical trials [22].

**Fig. 1:**
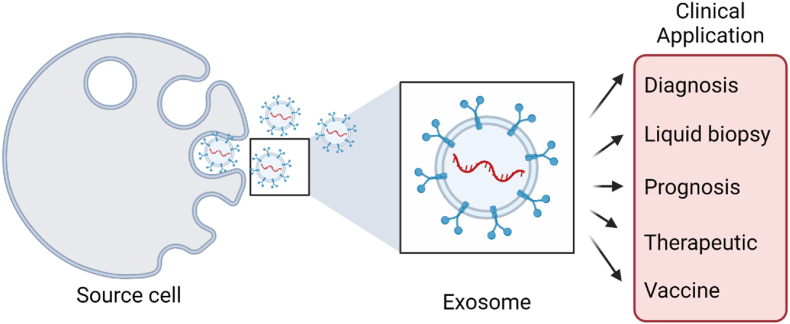
**Clinical applications of exosomes.** Exosomes can be used for clinical purposes, such as biomarker analysis-based diagnosis, liquid biopsy, prognosis, therapy, drug delivery, and vaccine development.

The regulations consider the testing of samples such that it does not have any contamination (microbial and viral), and this is a must requirement standard that needs to be addressed during the manufacturing and quality control related to therapeutics; this also oversees the performance of clinical trials. Overall, it is important to establish regulations to ensure that EVs are used most effectively and safely to realize this innovative therapy's full potential. Through regulations and standards, there is potential for the clinical implementation of exosomal therapy to become a reality and revolutionize how we diagnose and treat diseases [16,17,22]. Further clinical applications and diversification of exosomes have been summarized in Fig. 1.

## 2 Classification of EV production

Exosome production must be monitored following the regulations set by the Center for Biologics Evaluation and Research (CBER) as biological substances [17]. This means the regulatory framework for products in the biological substance category applies to exosomes, regardless of the individual types. Thus, the supposedly anti-tumor vaccine incorporating exosome usage will come under regulations that should be taken for manufacturing therapeutic cancer vaccines [23]. To determine the medicinal type of exosomes, the ISEV assesses the functional moiety of EV-based therapy and defines these therapies under biological medicines sections. This is defined majorly by four categories: (i) therapeutics that are acquired from unmodified cells and cell lines; further, (ii) therapeutics that are acquired by genetically modifying cells (without the usage of trans-gene); followed by this is, (iii) therapies known as gene-based therapy products (GTP) which are as acquired from either exosomes or genetically modifying cells using trans-gene. Further additions in this classification are, (iv) drug-delivery systems, in which exosomes are used as either biological or chemical components; these systems are known as biological medicine.

A further addition was made in 2007, which stated that all biological medicinal products were defined as Advanced Therapy Medicinal Products (ATMPs). ATMPs were further subdivided into categories based on their biological, physiochemical, and immunochemical properties. This subdivision compromises somatic cell-based therapies, gene-based therapies, and products generated from tissue engineering.

In conclusion, exosomes must be regulated according to the regulatory framework for biological substances and classified as ATMPs. This classification is based on their functional moiety and biological, physiochemical, and immunochemical properties [17,24,25].

## 3 Safety profile, manufacturing & standardization of EVs based therapeutics

EVs are promising therapeutics currently being explored for their potential to treat various diseases and conditions [26]. Adopting EVs in clinical practice faces several technical challenges, including heterogeneity in EV populations and cargo, which complicates standardization and efficacy assessment. Additionally, scaling up production while ensuring quality control is difficult due to varying isolation methods and storage conditions. Key analytical approaches include ultracentrifugation for purity, size exclusion chromatography for scalability, and electroporation for efficient cargo loading, each with specific advantages and limitations [27,28]. As with any therapeutic, EVs' safety profile, manufacturing, and standardization must be considered for clinical translation. The mechanism of action (MoA) of an EV-based therapy is essential for clinical translation. In order to have a clinical-grade product, important points to consider are having a better knowledge of active substances to be used in EVs, and the properties of active substances, followed by quality control measures taken during the processing of a clinical-grade product [29,30]. Pre-IND studies and animal models can help to verify the efficacy and safety of an EV-based therapy, reducing the risk associated with its use in a clinical setting. To commercialize and scale up of manufacturing process for EV-based therapeutics, a vigorous quality administration, technologically superior and up-to-date facility that complies to GMP must be in place [25,31]. The primary goal is to ensure the donor and patient's safety.

**Fig. 2:**
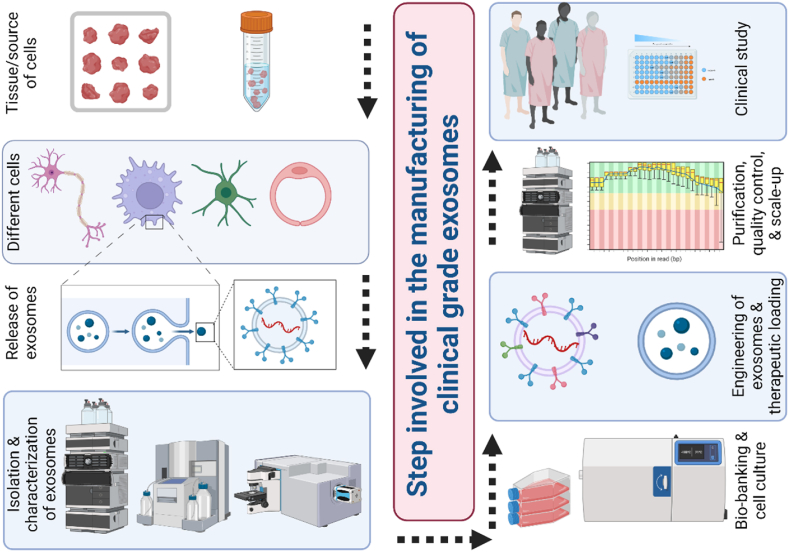
**Manufacturing and validation of exosomes for clinical purposes.** Manufacturing and validation of exosomes for clinical purposes involve multiple steps. First, the isolation of exosomes from different cell types and characterization must be done. Then, bio-banking and cell culture are used to engineer exosomes and drug loading. Purification and quality control should be performed, followed by scale-up. Finally, a clinical study using the validated exosomes should be conducted to ensure safety and efficacy. All these steps are necessary to manufacture and validate exosomes for clinical use successfully.

Additionally, an optimized protocol for EV isolation followed by storage must be established to ensure consistency and quality of the EV-based product. This includes reagent standardization, storage container selection, and storage time requirement and procedure [32]. This requires implementing a quality management system, technologically superior, and up-to-date facility that complies with GMP. Clinical translation can be achieved by taking the necessary steps to ensure the safety and efficacy of an EV-based therapy [12]; this is summarized in Fig. 2.

There have been different guidelines depending upon the country of EV manufacturing. However, The International Council for Harmonisation of Technical Requirements for Pharmaceuticals for Human Use (ICH) decides and defines various parameters for EVs manufacturing, storage, purity, and processing [[33], [34], [35]]. Various guidelines which are essential for early-stage development and manufacturing of EVs are follows.

1) Applications of therapeutic is for which type of population (small population/rare disease or large population/broad market). In case of existing or broad market usage is there any existing therapeutics is there and if yes one can compete with them or design a new one or design of a new therapeutics is always highly appreciated.
2) Large scale -manufacturing needs to be check and evaluated for reproducibility and quality insurance for each batch size. The storage of master and working cell banks, along with media type need to be classified.
3) Route of application and single or conjugated form application to the patient needs to be defined. Along with this risk-based approach in the case of human originated EVs must be classified and ruled out.

However, even after all this the major influence on EV manufacturing will be source of choice for the manufacturing. Majority of EVs are reported from human origin and derived from source such as blood/plasma, mesenchymal stem cells (MSCs), induced pluripotent stem cells (iPSCs), HEK293 (human embryonic cells line) and CAP (CEVEC Amniocyte Production cells, CEVEC Pharmaceuticals GmbH) [[36], [37], [38], [39]]. Moreover, EVs from engineered cell lines are preferred as they provide a better opportunity for scale up and reproducibility [40]. Apart from this non-human source such as plants and marine microalgae is also having a significant source of attraction [[41], [42], [43], [44], [45]].

Further upstream and downstream processing is also involved depending upon the EV source. For examples in case of natural sources such as human plasma/blood, plants, and bovine milk no upstream processing is required [46]. However, in case of primary/immortalized human cell lines such as MSCs for tissues regeneration requires the upstream processing as they are adherent cell lines/anchorage dependent cell lines [44,47,48]. To increase the bio-reactor cell densities, 3D bioreactors with serum free media are preferred so that this will increase the purity, reproducibility and lower the cost of performance. On the other hand, established cell lines such as HEK293 or CAP which can grow easily in suspension use less upstream processing [46,49]. As these cell lines can grow easily in advance bio-reactors type (Fed-batch or high-density processing reactors) with defined media and reproducibility [50,51]. Similarly, in engineered or WT microbial cells can be used to for EV production using this kind of bioreactors. However, market demands for such EVs are limited which limits their production on large scale despite the fact of their low-cost production [52,53].

As upstream is decided by the source downstream is govern by the applications of those EVs. Down-stream processing defines the purity of EVs, this is optimized and standardized by different parameters such as operating using, size of reactors and market need of the EVs [53,54]. However, in general steps involved in this are cell separation through differential centrifugation, which is a slow and laborious method, followed by filtration. Due to this it is a very time-consuming step for large scale manufacturing of EVs [[55], [56], [57]]. Newer methods for cell separation and filtration such as chromatographic separation based on ion-exchange, affinity chromatography, & hydrophobic interaction, tangential flow filtration, depth filtration and dead-end filtrations are used, respectively [[58], [59], [60]]. Apart from these newer purification methods such as nanoscale displacements, field flow fraction and affinity separation through desired ligands are in deployment for use [57,59,61]. These techniques can yield 10^17^ and 10^18^ EVs from a batch of 500L of starting material which can be optimized and scaled up in future.

## 4 Different cells derived EVs and their clinical applications

As we all know, EVs from various cell types have different characteristics, and because of this, we can use EVs from different cell types for different clinical applications [[62], [63], [64]]. Thus, EVs from different sources have different and unique properties and signaling molecules [64]. They possess a broad spectrum for exploring their role in clinical applications [65]. EVs from MSCs play the main role in replacing dead tissue and differentiation into desired cell types during any injury [[66], [67], [68], [69]]. This is done by EVs present in the secretome of MSCs leads to the mechanism mentioned above, as it has been reported that about 1 % of transplanted MSCs reaches the site of injury during healing [[70], [71], [72], [73]].

Further, it has been reported that all the MSCs from different sources (such as bone marrow, umbilical cord, and adipose tissue) can produce EVs [[74], [75], [76], [77]]. EVs produced from stem cells isolated from bone marrow (BMSC-EVs) help inhibit TNF-α mediated inflammatory pathways and improve osteoarthritis [78]. Similarly, it has been observed in another study that administrating BMSC-EVs increased the tendon population and decreased apoptotic cells and inflammatory cells, which in turn recovered tendon damage [79]. Another study suggested the effectiveness of BMSC-EVs on kidney issues [80]. A similar effect is seen in recovery from myocardial infarction [81,82], and they can increase the life span in-vivo [83]. Apart from this, the ability of BMSC-EVs to alleviate inflammation can be used as a promising strategy for COVID-19 treatments [[84], [85], [86]]. EVs derived from stem cell of umbilical cords (UCSC-EVs) has also been shown to have an impeccable effect, EVs from UCSC has been shown to improve motor functions and nerve regeneration in damaged areas [87]. It has been shown as an effective agent in liver fibrosis [88] by reducing collagen deposition, inflammation reduction, apoptosis, and oxidative stress [88]. It has also been found to improve BMSCs differentiation into osteogenic through microRNA-3960 [89]. A similar effect has also been observed in kidney disease [90]. However, EVs from neither BMSC nor UCSC has been shown to have similar therapeutic effect; the only areas where UCSC have a higher impact than BMSC are when it comes to prenatal injuries and gynecological disorders [91,92].

Moreover, EVs secreted from human umbilical cords have better potential as therapeutics than EVs from other sites as they have higher proliferation rates [93]. Another source of EVs secretion is adipose tissues, the effectiveness of adipose tissue stem cell-derived (ADSC-EVs). In a study, it has been observed that ADSC-EVs help reduce interlukin (IL)-5 levels in the lungs further decreasing allergic asthma reactions [94]. ADSC-EVs are found to be effective in Huntington disease (HD) [95] and Alzheimer disease (AD) [96]. When injected in mice, it has been observed that lesser spinal cord inflammation and demyelination [97]. Further, ADSC EVs are effective in wound healing and scar formation [97,98]. They also play an important role in modulating gene expression and cytokine release patterns, which will decrease IL-1β-mediated inflammation [99].

Another great source of EVs is from blood, and they are of great interest due to their vast applications [[100], [101], [102], [103], [104]]. They are known as blood cell-derived EVs (BC-EVs). As they are easily accessible and can be harvested at a lower cost, scaling up is very convenient [105]. The great EV source is derived from red blood cells (RBC-EVs). They are abundant and abundant with an extended life span [106]. RBC-EVs lack intracellular DNA [106]; EVs which are derived from mature RBCs they don't have mitochondrial and nuclear DNA [107]. Apart from this, one can retrieve around 5 billion RBCs/mL of human blood, which can be further used for EVs generation [107].

Moreover, the availability of such high cell numbers from such low amounts can rule out the addition of any mutation during cell passaging for higher cell number generation [105]. Thus, they can easily prevent immune clearance and be an important tool in cancer diagnostics [108]. Multiple studies have proved the therapeutic efficacy of RBS-EVs in tumor suppression, such as breast tumor growth [109], myeloid leukemia cell growth [109], and lung carcinoma [108]. However, higher concentration RBC-EVs can lead to coagulations, oxygen homeostasis, nitric oxide dysregulation, and thrombosis. Thus, further in-depth investigation is required to evaluate EVs' role in therapeutics [108]. Other than RBC, platelet-derived EVs (P-EVs) are another source of EVs from blood, and around 12k EVs derived from platelets can be harvested from one 1 μL of healthy plasma [110]. P-EVs are secreted at a higher concentration during inflammations and chronic diseases. Thus, extracting and modifying them for therapeutic purposes can be achieved easily [[111], [112], [113], [114], [115], [116]]. P-EVs are shown to affect tissue healing, wound repair, and scar removal [104,117]. It has been reported that P-EVs can induces wound healing mediated through PI3K-Akt/MAPK-Erk pathways and via yes-associated protein (YAP) activation [104]. In the rat model, it has been reported that P-EVs can decrease disease scarcity in osteonecrosis and diabetes [104,118]. However, as in the case of RBC-EVs, P-EVs need to be expanded further so that one can use their potential to the fullest.

Another source of EVs is immune cell-derived EVs (ICD-EVs); in the case of ICD-EVs, they can induce either response (Immune-stimulatory/Inhibitory) on the basis of cell type and parental properties [119]. Thus, because of this, they can play roles in both physiological/pathological processes. ICD-EVs from immune cells, such are macrophages (Mɸ), T-cells, Dendritic cells (DCs), and natural killer (NK) cells, have different and individual features/functions distinct from one another [[120], [121], [122]]. It has been observed that both mature and immature DCs produces different responses and activates T-cell responses and tolerogenic pathways, respectively [122]. Thus, these ICD-EVs have a vast ability to be used as a therapeutic agent. EVs isolated from DCs of lung and metastatic melanoma cancer have proven to activate immune response [123,124]. Further various modification for co-delivery has been used, leading to anti-tumor activities [121]. Additionally, DCs-derived EVs are useful in cardiac injuries [125,126], against HIV-1 infection. Similarly, T-cell-derived EVs are reported to be active against tumors [127] and transplantation tolerance [128]. NK cell-derived EVs have had a similar effect against tumors [129]. ICD-EVs have the property to act as immune-suppressing and modulating, which makes them a favorable candidate for therapeutic purposes.

Another set of fascinating EVs derived from neural cells (N-EVs), such as neurons, microglia, oligodendrocytes, and astrocytes, plays a vital role in communications and can play a vital role significant role in central nervous system diseases [130,131]. N-EVs can be isolated from biofluids and can act as a biomarker for neurodegenerative disease diagnosis and prognosis [132,133]. It has been reported in a study that N-EVs are found to reduce toxic fibrils and ameliorate Aβ pathology in mice [134]. It has also been useful in strokes [135,136] and Huntingtin disease [137]. Some of the recent EVs trails has been summarized in tabular form in Table 1.

**Table 1:** Clinical trials for exosomes as biomarkers or therapeutics for different studies.

Diseases	Sub-type		Year	Application	Country	Clinical Trail File number	No of patients Originally enrolled	Methodology used	Clinical Aim
Cancer	Breast		2011	Theranostics	United States	NCT01344109	100	Serum-Plasma Centrifugation	To evaluate the use of tumor-derived exosomes as a marker for response to therapy in neoadjuvant chemotherapy for newly diagnosed breast cancer.
		HER-2 positive	2019	Diagnostic	United Kingdom	NCT04288141	40	FLIM-FERT	To evaluate the expression of HER2-3 in samples from patients receiving HER-2 targeted therapy.
		Leptomeningeal	2019	Diagnostic	France	NCT03974204	0	Proteomics	To investigate the association between initial and after proteomic profile of cerebrospinal fluid in breast cancer patients.
	Gastric		2013	Diagnostic/Prognostic	China	NCT01860118	95	Proteomics	To investigate biomarkers associated with Parkinson's disease.
	Brain Tumor		2012	Diagnostic	United States	NCT01550523	13	MRI-based Radiographic responses to treatment	Induce apoptosis at the tumor site using the patient's cells after treatment.
	Prostate		2014	Diagnostic	United States	NCT02702856	2000	Gene expression analysis	Validation of urinary exosome gene signature in men showing symptoms of prostate cancer.
			2016	Diagnostic	Switzerland	NCT03034265	24	Proteomics	To determine the biomarkers for prostate cancer.
			2019	Prognostic	United Kingdom	NCT04167722	100	Laboratory culture conditions	To study the samples of fat tissues and prostate tissue for participants undergoing radical prostatectomy.
			2020	Diagnostic	United States	NCT04357717	150	Laboratory culture conditions	To evaluate the ExoDx prostate test in repeating prostate biopsy.
			2021	Diagnostic	United States	NCT03608631	15	Laboratory culture conditions	To evaluate the effect of exosomes on the dose of pancreatic cancer.
	Pancreatic		2015	Diagnostic	United States	NCT02393703	111	ECM using IF and IHC	To analyze and isolate exosomes from blood and tissue from pancreatic cancer.
			2017	Diagnostic	France	NCT03032913	52	Biopsy and Flow cytometry	Blood samples for cancer and exosomes from them.
			2018	Diagnostic/Prognostic	China	NCT03821909	30	Endoscopic ultrasound (EUS)	Exosomes obtained from portal venous blood in pancreatic cancer patients are further used as prognostic markers.
		Non-tumorous small cells	2010	Diagnostic	France	NCT01159288	41	Laboratory culture conditions	Exosomes from dendritic cells treatment with tumor induction chemotherapy.
			2017	Diagnostic	China	NCT03830619	1000	Laboratory culture conditions	Serum of exosomes noncoding RNA as a biomarker for diagnosis of lung cancer.
		Metastases, osteosarcoma	2017	Diagnostic/Prognostic	China	NCT03108677	90	Laboratory culture conditions	Circulating exosome RNA as a biomarker in lung metastases of primary high-grade osteosarcoma.
		Early	2018	Diagnostic	China	NCT03542253	80	CT-Scan	Exosomes from cancer tissue and para-cancerous tissue.
		Non-tumorous small cells	2020	Prognostic	China	NCT04427475	200	Plasma	Exosome levels after and before immunotherapy.
	Thyroid		2016	Prognostic	Taiwan	NCT02862470	22	Laboratory culture conditions	Estimation of biomarkers after the treatment.
			2018	Prognostic	Taiwan	NCT03488134	74	Proteomics	Estimation of biomarkers after the treatment.
Cardiovascular disease	Hemodynamic instability		2017	Diagnostic	Taiwan	NCT03267160	30	Proteomics	Analyzation of autophagy and apoptosis-related biomarkers.
	Atrial fibrillation		2018	Diagnostic	Israel	NCT03478410	35	Biopsy	Role of exosomes isolated from epicardial fat in atrial fibrillation.
	Myocardial Infraction		2019	Diagnostic	China	NCT04127591	10	Gene expression study	Exosomes from the peripheral blood of patients.
	Prehypertension		2020	Diagnostic	United States	NCT04142138	9	Mass Spectrometry	To analyze the changes in urine electrolytes and exosome protein abundance patterns during nutritional changes.
Obstetrics & Gynecology	Preeclampsia		2016	Diagnostic	Cairo, Egypt	NCT03562715	200	Laboratory culture conditions	Blood samples from pregnant women were collected, and exosome isolation was done.
			2020	Diagnostic	United States	NCT04154332	64	Laboratory culture conditions	Exosomes from blood samples normal and pathological conditions for biomarker identification.
	Endometrial Fluid		2016	Diagnostic	Spain	NCT02797834	300	Mass Spectrometry and NGS techniques	Exosomes are secreted by human endometrium to the endometrial fluid.
	Oocyte		2020	Prognostic	Hong Kong	NCT04382872	60	Transcription studies	Exosomes isolated from young and aged women during oocyte maturation.
	Normal cellular metabolism		2016	Diagnostic	United States	NCT02748369	17	NMRS	In vivo assessment of TCA cycle flux in humans.
Neurodegeneration	Parkinson's disease		2013	Diagnostic	United States	NCT01860118	601	Proteomics	Exosome from Parkinson's diseases for biomarkers assessments.
	Cerebrovascular Disorders		2017	Diagnostic	Iran	NCT03384433	5	Proteomics	MSCs derived exosomes for acute ischemic stroke.
	Alzheimer Disease		2020	Diagnostic	China	NCT04388982	9	Proteomics	Exosomes derived from adipose mesenchymal stem cells for Alzheimer's disease.
Infectious disease	COVID-19		2020	Theranostics biomarker	Turkey	NCT04389385	60	Gene expression study	To treat coronavirus pneumonia using exosomes derived from allogenic COVID-19 T cells.
					Russian	NCT04602442	90	Proteomics	To treat two-sided pneumonia using exosomes isolated from COVID-19.
						NCT04491240	30	Gene expression study	Exosome inhalation of the patients hospitalized with novel coronavirus pneumonia.
					China	NCT04276987	24	Gene expression study	MSCs derived exosomes for treatment with coronavirus pneumonia.
						NCT04313647	24	Gene expression study	MSCs derived exosomes in COVID-19 infection.
					United States	NCT04623671	63	Gene expression study	Intravenous infusion of CAP-1002 in patients with COVID-19
						NCT04384445	20	Proteomics	To investigate the potential efficacy of HAF-derived acellular products in subjects suffering from COVID-19 infection.
						NCT04657406	20	Bioinformatics	Biomarker assessments for patients with mild to moderate COVID-19.
						NCT04798716	40	Gene expression study	MSCs derived exosomes from COVID-19.
			2021		Israel	NCT04747574	35	Gene expression study	Evaluation of CD-24 exosomes in patients suffering from moderate to severe COVID-19 infection.
Miscellaneous	Acute Respiratory Distress Syndrome		2020	Theranostics	China	NCT04602104	18	Gene expression study	MSCs derived exosomes for treatment of acute respiratory distress syndrome.
	Bronchopulmonary Dysplasia		2021	Theranostics	United States	NCT03857841	3	Gene expression study	BMMSCs derive extracellular vesicles in the treatment of bronchopulmonary dysplasia
	Pulmonary infection-drug resistance		2020	Theranostics	China	NCT04544215	60	Proteomics	Human-AMPCs derived exosomes for treatment of carbapenem-resistant gram-negative bacilli-induced pulmonary infection.
	Dry Eye		2019	Theranostics	China	NCT04213248	27	Laboratory culture conditions	MSCs derived exosomes for dry eye treatment in chronic graft versus host diseases.
	Multiple Organ Failure		2020	Theranostic	China	NCT04356300	60	Proteomics	Exosomes from MSCs for multiple organ dysfunction syndrome after aortic dissection.
	Macular Holes		2018	Theranostics	China	NCT03437759	44	Fundoscopy and physical examination	Healing of large and refractory macular holes using exosomes from MSCs.
	Sepsis		2016	Prognostic	China	NCT02957279	50	Laboratory culture conditions	Impact of peripheral blood dendritic cells-derived exosomes for prognosis in human sepsis.
	Diabetic retinopathy		2018	Prognostic	China	NCT03264976	200	Laboratory culture conditions	Serum exosomes miRNA and its function in the pathogenesis of diabetic retinopathy.
	Kidney transplantation		2018	Theranostics	France	NCT03503461	67	Laboratory culture conditions	Exosomes for kidney transplantation patients.
	Thyroid diseases		2019	Prognostic	China	NCT03984006	5	Thyroid ultrasonography	Detection of thyroid heart disease through urinary exosome proteins.
	Postoperative delirium		2020	Diagnostic	China	NCT04421872	1000	RNA and Protein assays	To analyze the disorder of the circadian clock gene and early cognitive dysfunction after general anesthesia.
	Diabetes Mellitus Type 1		2014	Theranostics	Cairo, Egypt	NCT02138331	20	Gene expression study	Cell-free blood-derived microvesicles for diabetes mellitus
	Blood Coagulation		2015	Prognostic	Germany	NCT02594345	18	FACS and Thermoelectrometry	Impact of exosomes derived from RBC on platelet function and coagulation in healthy humans.
	Ulcer		2015	Diagnostic	Japan	NCT02565264	5	Patch implementation around the wound	Impact of plasma-derived exosomes on wound healing.
	Dystrophic Epidermolysis Bullosa		2019	Diagnostic	United States	NCT04173650	10	Patch implementation around the wound	MSCs derived exosomes for the treatment of dystrophic epidermolysis wounds.

## 5 Conclusion and future Outlook

Exosome-based therapy is a novel platform that has the potential to revolutionize clinical care in a variety of fields. Exosomes, which are small vesicles released by cells, have been found to have immunomodulatory properties, making them a promising platform for diagnosing and treating various conditions. Exosomes have been shown to have stupendous impacts on the diagnosis and prognosis of conditions in disease of both infectious and non-infectious (such as autoimmune/neurodegenerative disorders and orthopedic/cardiovascular diseases), along with cancers [138,139]. The circulating pool of exosomes can be used as biomarkers for the early diagnosis of complex diseases in the blood [[140], [141], [142]].

Exosomes derived from MSCs can also be used for tissue repair and regeneration [143,144]. Exosomes have also been found to be anti-inflammatory and used in treating various inflammatory disorders [139]. Furthermore, exosomes have been studied for their potential to be used as vehicles for drug delivery in the case of anti-tumor therapies and pathogen vaccination. The novel potential of exosomes has led to increased research into the field and spurred the development of exosome-based therapies. This has resulted in the emergence of start-ups with proprietary technologies to unlock the full potential usage of exosomes, such as Capricor Therapeutics. Capricor therapeutics led to the development of exosomal therapeutics from cardio-sphere-derived cells (CDCs) known as CAP-2003. This technology is currently in the pre-clinical testing phase and is being evaluated for its pro-angiogenic, anti-inflammatory, and antifibrotic/antiapoptotic effects. Apart from CAP-2003, it has been shown to have the potential to be used in trauma-related injury treatment [145]. The application of exosomal therapy is vast, and continual breakthroughs in the field can generate novel therapies which will change the global face of pharmaceuticals. Exosomal therapy has gone from being a subject of academic interest to a future potential application [146]. Apparently, by seeing the capabilities of exosomes, it can be envisioned that exosome will leave a mark as an important theranostic tool for the diagnosis and therapeutics of various diseases [19].

## Informed consent statement

Not applicable.

## Authors’ contribution

AT conceived the outline. AT and DR wrote the manuscript. AT prepared the figures using Biorender. AT revised and edited the manuscript. All the authors agreed to the final version of the manuscript.

## Institutional review board statement

Not applicable.

## Data availability statement

Not applicable.

## Ethical approval statement

No ethical approvals or patient consent were necessary for the study.

## Funding

This work received no external funding.

## Declaration of competing interest

The authors declare that they have no known competing financial interests or personal relationships that could have appeared to influence the work reported in this paper.
